# Improved Prognostic Prediction of Glioblastoma using a PAS Detected from Single-cell RNA-seq

**DOI:** 10.7150/jca.44034

**Published:** 2020-04-06

**Authors:** Hongwei Liu, Qi Yang, Yi Xiong, Zujian Xiong, Xuejun Li

**Affiliations:** 1 Department of Neurosurgery, Xiangya Hospital, Central South University, Changsha, Hunan, 410008, China; 2 Hunan International Scientific and Technological Cooperation Base of Brain Tumor Research, Xiangya Hospital, Central South University, Changsha, Hunan, 410008, China; 3 Xiangya Medical School, Central South University, Changsha, Hunan, 410008, China

**Keywords:** glioblastoma, single cell, differentially expressed genes, survival analysis, prognostic model

## Abstract

Glioblastoma (GBM) is a common malignant brain tumor of the central nervous system with a poor prognosis**.** In order to identify the prognostic signatures of GBM, we screened differentially expressed genes (DEGs) that were based on a single-cell RNA sequencing (scRNA-seq) dataset. These genes characteristically represent the intra-tumor heterogenicity of glioblastoma. Moreover, we performed univariate analysis, log-rank test and multivariate Cox regression analyses to confirm a gene set that could be related to the overall survival (OS) among DEGs. Prognostic associated signatures (PAS) were utilized to construct a model for predicting OS in GBM patients. When considering either the training or the validation sets, time-dependent receiver operating characteristic (ROC) curves all indicated that our model displayed an excellent predictive ability. Additionally, we analyzed PAS at the single-cell level and found that the PAS score was associated with somatic mutations and clinical factors. Three factors, which included the PAS score, radiotherapy status, and age, were all used to establish a nomogram to predict the 6-month and 1-year survival probabilities. In conclusion, we constructed an optimal model that was derived from scRNA-seq to better predict the survival probability of GBM patients. These genes might also act as potential prognostic biomarkers and enable surgeons to develop individually therapeutic schedules and improve the prognosis of GBM patients.

## Introduction

Glioblastoma (GBM) is the most common malignancy among primary central nervous system (CNS) tumors, which is classified as a grade IV glioma according to the World Health Organization (WHO) 1. The median overall survival (OS) of GBM patients remains at only 15 months post-diagnosis, with a 5-year disease-free survival probability of 10 percent 2-4. The poor-prognosis is mostly due to a high proliferation rate, treatment-resistance to chemotherapy and targeted therapies, and aggressive infiltration of cancer cells into the surrounding normal brain tissues 5.

Over the past few decades, microarray analysis and RNA sequencing (RNA-seq) from bulk tissue has evolved as an important tool in the analysis of differential gene expression across the transcriptome 6. With the development of this technology, many public cancer databases that are associated with various omics approaches have been established to reveal the underlying mechanisms accounting for disease occurrence, which includes the Cancer Genome Atlas (TCGA) 7 and the Gene Expression Omnibus (GEO). Using these gene expression profiles to identify biomarkers associated with prognosis, subsequent studies are increasingly being reported. However, GBM is a highly heterogeneous cancer wherein differential genes that are screened from bulk tissue data are not sufficiently representative of this disease 8.

Recently, single-cell RNA-seq (scRNA-seq) analysis has gradually emerged as a new area of intense research effort with its advances helping to explain intra-tumorigenic heterogeneity 9-11. In addition, tumor tissue that is resected from the core tumor of affected patients might include different cell types that harbor distinct phenotypic states 12. Single-cell RNA-seq technology enables scientists to identify individual cells in heterogeneous cell populations, which can overcome the aforementioned limitations 13, 14. Moreover, scRNA-seq analysis enables the discovery of significant genes that are truly characteristic of tumor cells 15.

In this current study, we first identified differentially expressed genes (DEGs) from a scRNA-seq dataset of tumor cells as compared normal cells. These genes characteristically represent the intra-tumor heterogenicity of glioblastoma. Then we integrated the dataset with the bulk RNA-seq dataset from the TCGA and microarray datasets of GEO to obtain a prognosis-associated gene set. Finally, we explored utilizing this gene set to construct a more effective model to accurately predict the prognosis of GBM patients. This approach has the potential of providing guidance for additionally targeted and individualized treatment of patients.

## Materials and Methods

### Data sources

Raw sequencing data of single-cell datasets in this study was fetched from the Gene Expression Omnibus (GEO) database under the accession number GSE84465, for which, 3589 cells were included in the following analysis for which PAS scored an initial quality check. Raw fastq data was mapped to the hg19 human reference genome by HISAT2 (v2.1.0) 16 (with parameters: -k 10 -rdg 99999999, 99999999 -rfg 99999999, 99999999 -mp 1, 1 -np 1 -score -min L, 0, -0.1 -no-mixed -no -softclip -no -discordant -secondary -seed 12345), and aligned reads were quantified by feature counts software 17 to the count level.

**Figure 1:**
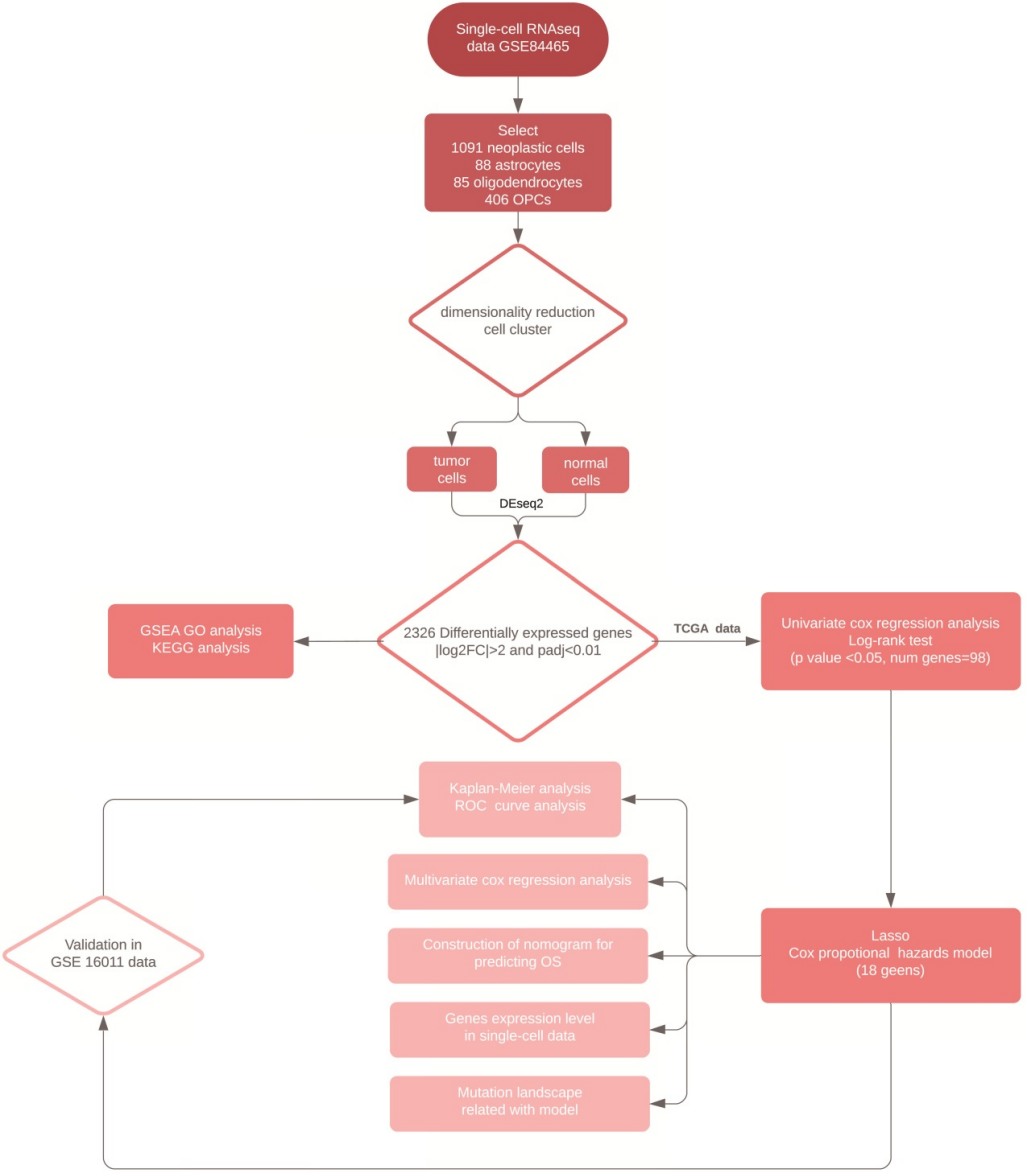
Workflow of the analytical procedure for constructing and validating prognostic signatures in GBM patients.

We downloaded the TCGA-GBM RNA-seq and clinical data as a training set from the University of California at Santa Cruz (UCSC) Xena Browser. The fragments per kilobase of exon per million fragments mapped (FPKM) value, was used to normalize gene expression from which, we transformed it into log2 (FPKM+1) datasets. The microarray data from the GSE16011 was also collected from the GEO database as a validation set and was processed by normalizing gene expression signals and log2 transformation through mas5 algorithm in R package “affy” (v1.64.0). In total, there were 154 GBM patients in the TCGA- GBM dataset and 155 GBM patients in the GSE16011 dataset with complete clinical data. In addition, the MuTect2 somatic mutation data was obtained from the UCSC Xena Browser. A workflow of the analysis conducted in this study was shown in Figure 1.

### Cell clustering in single-cell RNA-seq data

We used the statistical R package “Seurat” (v3.1.1) 18, 19 to process single-cell data. First, we normalized the data by following the standard pre-processing workflow in Seurat. Then, we calculated a subset of features that exhibited high cell-to-cell variation, from which we selected the top 500 features by setting the method as dispersion. Finally, the Uniform Manifold Approximation and Projection (UMAP) algorithm was used to perform dimensionality reduction. The same marker profiles of the cell type that were reported in the original paper 12 were compared with our current study to define the type of each cell.

### Differential expression analysis

Differentially expressed genes (DEGs) were identified with the R package “DESeq2” (v1.22.2) 20 by defining neoplastic cells as the tumor group, and then astrocytes, oligodendrocytes, oligodendrocyte progenitor cells (OPCs) as the normal group. The DESeq2 pipeline was used with default settings. We filtered genes with the criteria by a log2 fold change >2 and adjusted this to an alpha value of p <0.01.

### Functional and pathway enrichment analysis

To compare the biological processes (BP), cellular components (CC) and molecular functions (MF) of DEGs, we used the R package “clusterProfiler” (v3.10.1) 21 to conduct gene set enrichment analysis (GSEA) of the Gene Ontology (GO). The five most significant biological processes in the results were shown with an adjusted alpha value of p <0.05. Additionally, the Kyoto Encyclopedia of Genes and Genomes (KEGG) pathway enrichment analysis of DEGs was performed with the same package. An UpSet plot of the top 10 pathways were drawn from this research.

### Survival analysis

The log-rank test, univariate Cox regression analysis and multi-Cox regression analysis were respectively used to screen PAS with OS values using the R package “survival” (v3.1-8). The threshold with significance in all methods was set at an alpha value of p <0.05. In addition, Kaplan Meier (KM) survival curves were generated to graphically exhibit the prognostic outcomes between high and low risk groups that were divided through the median of gene expression levels or the PAS score.

### Construction of the prognostic model

Due to the high-dimension features and complexity of the data, we performed a least absolute shrinkage and selection operator (LASSO) method analysis using the R package “glmnet” (v2.0-18) 22. In addition, 10-fold cross-validation was used to establish a best Cox proportional hazards model. Then, to quantify the risk of OS for each patient, the PAS score was calculated as the sum product of the RNA (Expi) expression levels and LASSO coefficients (Li). The efficacy of the prognostic model was validated by depicting the areas under the curve (AUC) of the ROC by using the R package “survivalROC” (v1.0.3). To monitor the OS and to predict the survival probability in GBM patients, we merged the PAS score with interesting clinical variables and constructed a nomogram using the R package “regplot” (v0.2).

### Oncoprint of somatic mutation

Somatic mutation information was stored in Mutation Annotation Format (MAF) form and was visualized using the R package “maftools” (v1.8.10) tool 23. All parameters were set to the default settings.

### Statistical Methods

All data analyses were completed using the 'R” statistical software package (v3.5.3) and the corresponding fundamental analyses packages. An alpha value of p <0.05 was considered statistically significant.

## Results

### Differentially expressed gene identification from a single-cell RNA-seq dataset

**Figure 2:**
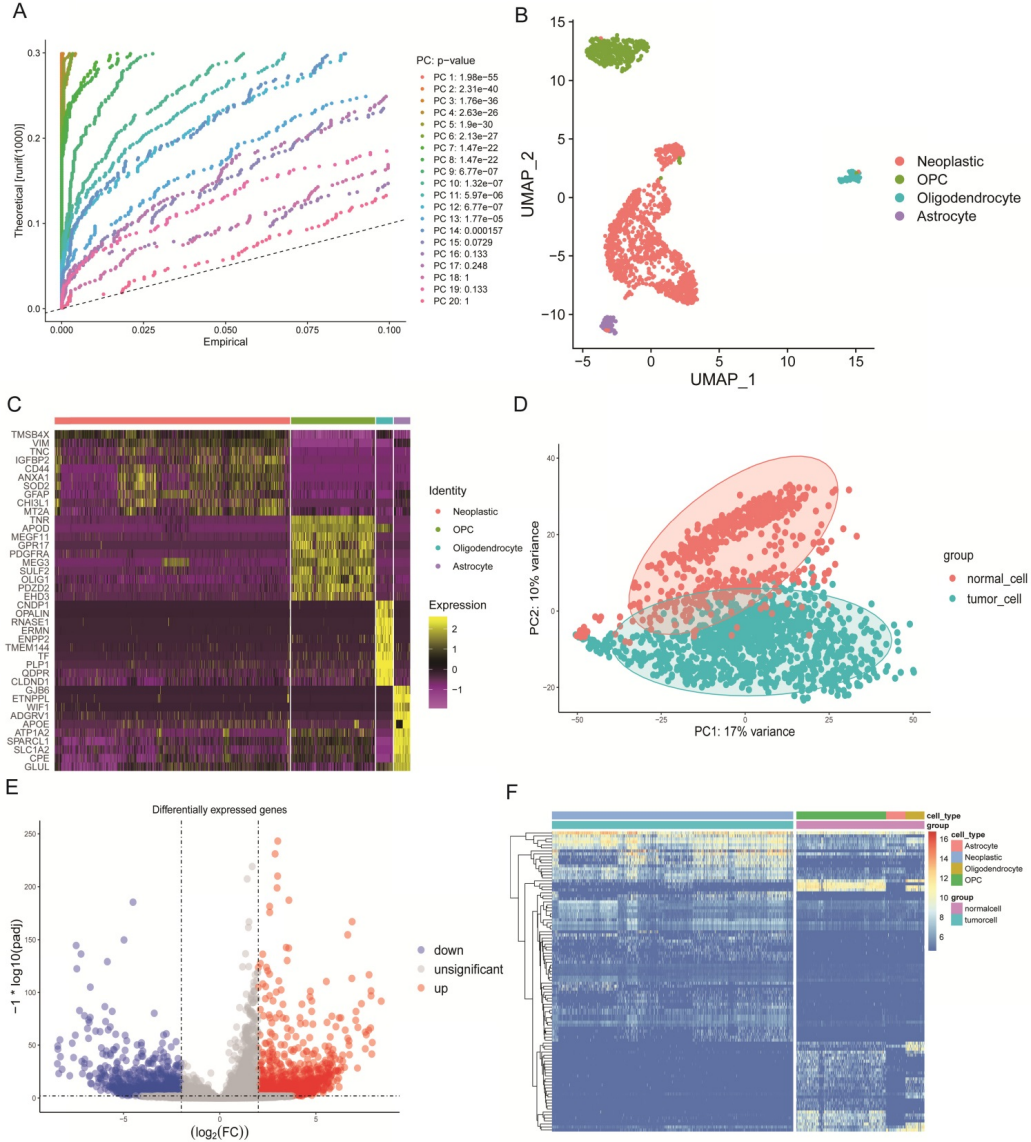
** Characteristics of single-cell RNA-seq data and DEGs. (A)** Jack Straw Plot showing the p-value distributions for each PC. **(B)** Dimension reduction analysis of single-cell RNA-seq data by the UMAP algorithm that clusters cells into four groups. **(C)** Heat map expression profiles of the top 10 genetic markers in each cluster. **(D)** PCA of tumorigenic and normal cells that can be clearly separated. **(E)** Volcano plot of DEGs with a log2 fold-change >2, and an adjusted alpha value of p <0.01. **(F)** Heat map expression profiles of the 100 most significant genes for tumorigenic and normal cells.

We selected 1670 single cells that included 1091 neoplastic cells, 88 astrocytes, 85 oligodendrocytes, and 406 oligodendrocyte progenitor cells (OPCs). These cells were analyzed by the standard workflow of the R package “Seurat.” A linear dimensionality reduction method helped identify significant components (Figure 2A). Additionally, we used the Uniform Manifold Approximation and Projection (UMAP) algorithm, which is a novel manifold learning technique for dimension reduction to visualize the single-cell RNA-seq data based on the significant components. We discovered that cells could be clustered into four groups (Figure 2B). The differential marker genes between clusters are illustrated in Table S1, and the top 10 most enriched genes in each cluster were exhibited in the heat map (Figure 2C). Next, we compared the marker genes in our study with those identified in the original article 12 and used the same marker genes to name the clusters, where results were similar.

Concrete classification information of cells used in this study are illustrated in Table S2. In order to find the DEGs between tumorigenic and normal cells, we defined astrocytes, oligodendrocytes and OPCs as normal cells. By principal component analysis (PCA), tumorigenic and normal cells were separated based on principle component 1 (PC1) and PC2 (Figure 2D). Then the “DEseq2” package identified the DEGs. We excluded genes with the cut-off criteria of log2 fold change >2 and adjusted the alpha value to p<0.01. In the results, we included the 1367 identified up-regulated genes and the 959 down-regulated genes in tumor cells that were displayed in the volcano plot (Figure 2E). The two groups were clearly discriminated by these 100 DEGs in the heat map (Figure 2F).

### GSEA of GO and KEGG pathway enrichment analysis in DEGs

**Figure 3:**
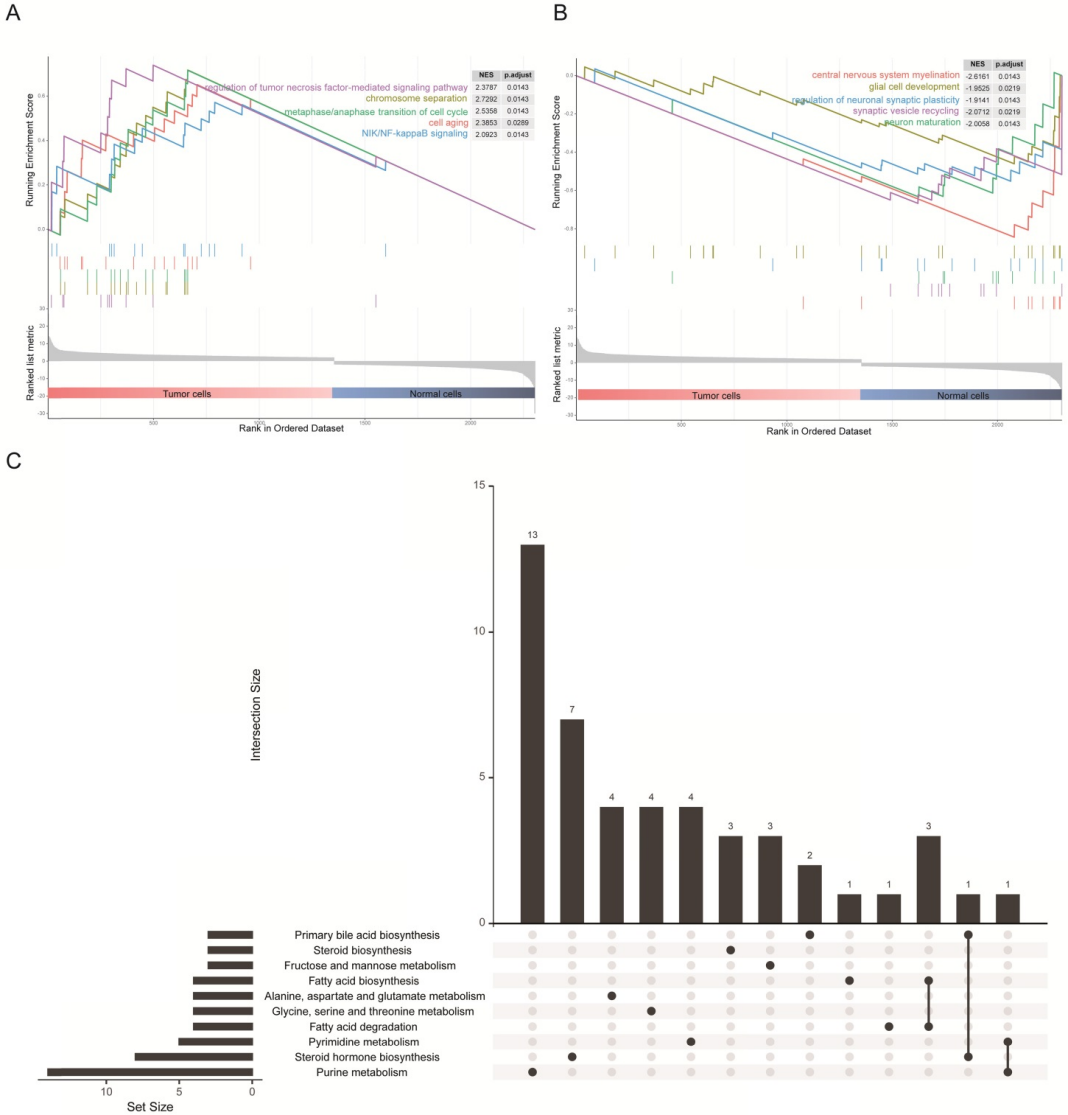
** GSEA results and KEGG analysis of differentially expressed genes. (A)** The five most significant biological processes for tumor cells in the GSEA of GO. **(B)** The five most significant biological processes for normal cells in the GSEA of GO. **(C)** Diagram illustrating the top 10 pathways in the KEGG enrichment analysis of DEGs.

The 1367 DEGs of the transcriptomic data from single cells were selected for gene set enrichment analysis (GSEA) to explore the biological effects. We chose the log2 fold change as the reference phenotype and the five most significant Gene Ontology (GO) biological processes were listed. We observed that up-regulated genes in tumor cells were enriched in the cell cycle, cell division process and tumor-associated pathways (Figure 3A), as for cellular components and molecular functions, these genes were enriched in the nuclear and DNA binding associated molecular process (Figure S1A and Figure S1B). By contrast, up-regulated genes in normal cells were enriched in glial cell development and synaptic transmission (Figure 3B), as for cellular components and molecular functions, these genes were enriched in cytoplasm and transmembrane transportation (Figure S1A and Figure S1B). These results were consistent with the characteristics of both cell types. In addition, it is intriguing to note that the KEGG pathway enrichment analysis revealed an association of DEGs with lipid and fatty acid metabolism (Figure 3C), and similar results were shown on enrichment analysis of just up-regulated or down-regulated DEGs (Figure S1C and Figure S1D). Precisely how an altered associated metabolism contributed to progression of GBM remains unknown. However, quite valuable research study outcomes have been published that illustrated how fatty acid synthesis was related to EGFR signaling in glioma stem cells 24.

### Construction of prognostic-associated signatures in the TCGA-GBM cohort

To discover prognostic-associated signatures, we combined the 2326 DEGs that were defined above with the TCGA-GBM bulk RNA-seq and clinical data. There were 154 GBM patients with intact clinical data and 110 genes of DEGs were not expressed in TCGA bulk RNA-seq data. Before constructing a Cox proportional hazards model to better predict OS in GBM patients, we used the log-rank test and univariate cox regression analysis respectively to preliminarily screen genes with prognostic potential.

**Figure 4:**
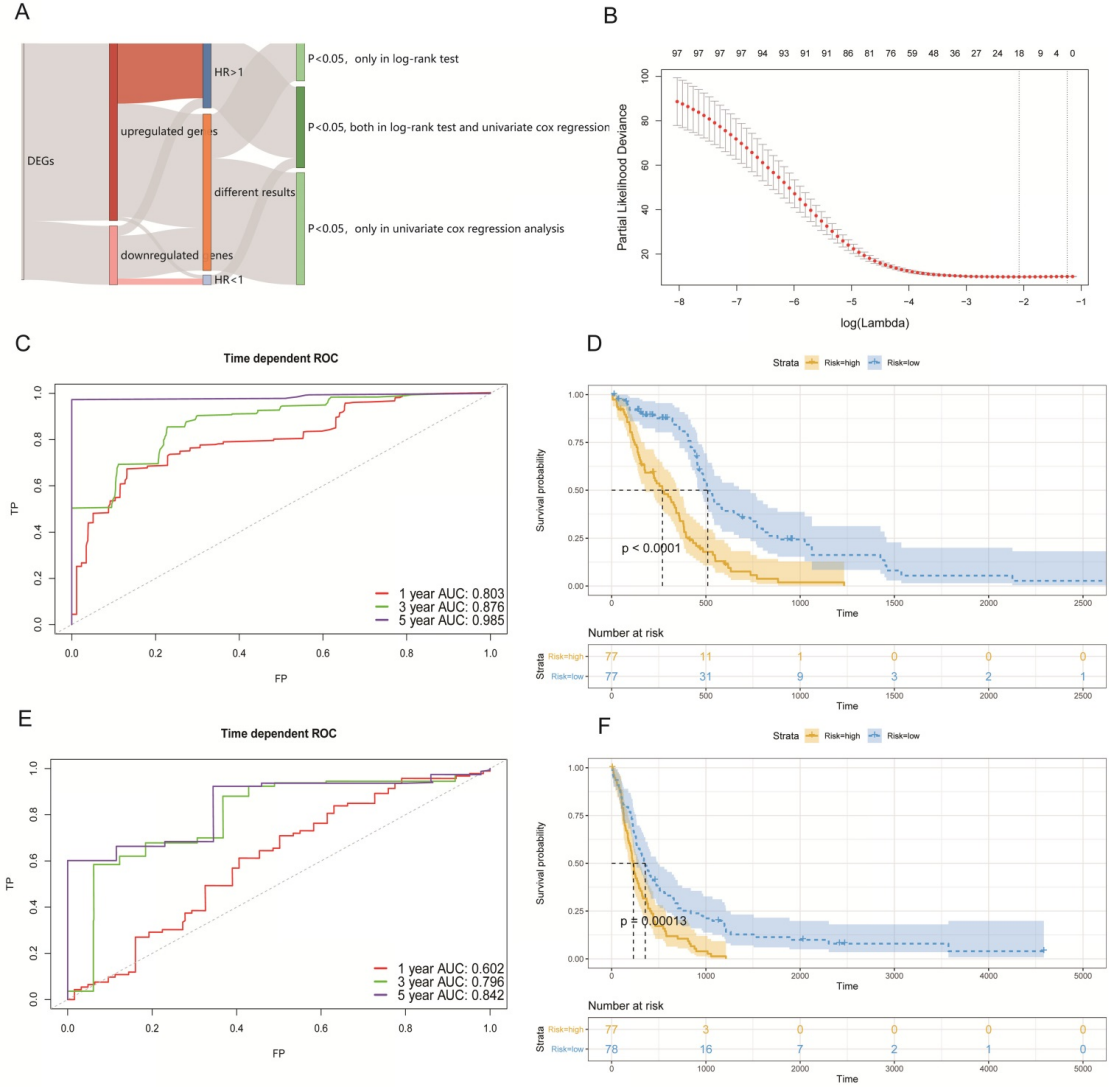
** Establishing a survival model in the GBM cohort. (A)** Summary of the selected DEGs with a prognostic capacity, in which we only selected genes during the area covered by indicated colors. **(B)** The optimal account of genes that corresponded to minimum lambda was 18 in the TCGA-GBM cohort.** (C)** The ROC analysis curves in predicting OS by the PAS score in the TCGA-GBM cohort. **(D)** The KM curves for low- and high-risk groups in the TCGA-GBM cohort. **(E)** The ROC curves for predicting OS by the PAS score in the GSE16011 cohort. **(F)** The KM curves for low- and high-risk groups in the GSE16011 cohort.

In the obtained results, we found that 181 genes had prognostic potential in the log-rank test and 279 genes had prognostic potential as determined by univariate Cox regression analysis (p < 0.05). We generally thought that the more up-regulated genes that were expressed in tumor cells of studied patients, then the worse their prognosis would be. Similarly, the greater the number of down-regulated genes that were expressed in tumor cells of studied patients, the better their prognosis would be. Thus, a total of 98 genes that included 89 up-regulated genes with a hazard ratio (HR) >1, and 9 down-regulated genes with a HR < 1 (p < 0.05; both in the log-rank test, and univariate cox regression analysis), were selected to construct a Cox proportional hazards model (Figure 4A).

By utilizing the least absolute shrinkage and selection operator (LASSO) method, we established a best Cox proportional hazards model (10-fold cross- validation) with 18 genes (Figure 4B). The complete information related to the OS of these 18 genes are shown in Table 1. In addition, Kaplan-Meier (KM) curves of each gene can be seen in Figure S2. The prognostic associated signature score (PAS score) for each patient was calculated as the sum of the product of the RNA expression levels (Expi) and LASSO coefficients (Li). In the TCGA-GBM cohort, the areas under the curve (AUC) of the receiver operating characteristic (ROC) curves of this model for predicting 1-, 3-, and 5-year OS were 0.803, 0.876, and 0.985 respectively (Figure 4C).

Furthermore, we divided patients into high-risk and low-risk groups according to the median of PAS score. KM analysis indicated that patients with a high PAS score suffered significantly poorer OS outcomes (p < 0.0001; Figure 4D). These results lend support to our model possessing good sensitivity and specificity.

### Validation of PAS in an external GBM cohort

To validate our PAS, we selected external data from GEO under accession number GSE16011 to determine whether it made sense in other cohorts. There were 155 GBM patients with complete survival data and microarrays. We conducted the same workflows to measure the AUC of the ROC curves and divided patients into two groups by the median of the PAS score. In the results analyses, the AUC of the ROC curves of this model in predicting 1-, 3-, and 5-year OS rates were 0.602, 0.796, and 0.842 respectively (Figure 4E). Further, KM analysis also indicated that patients with a high PAS score suffered significantly poorer OS outcomes (p < 0.001; Figure 4F). All results revealed that these PAS were important in GBM patients without an increased level of bias.

### PAS levels in a single-cell dataset that is correlated with somatic mutations

**Figure 5:**
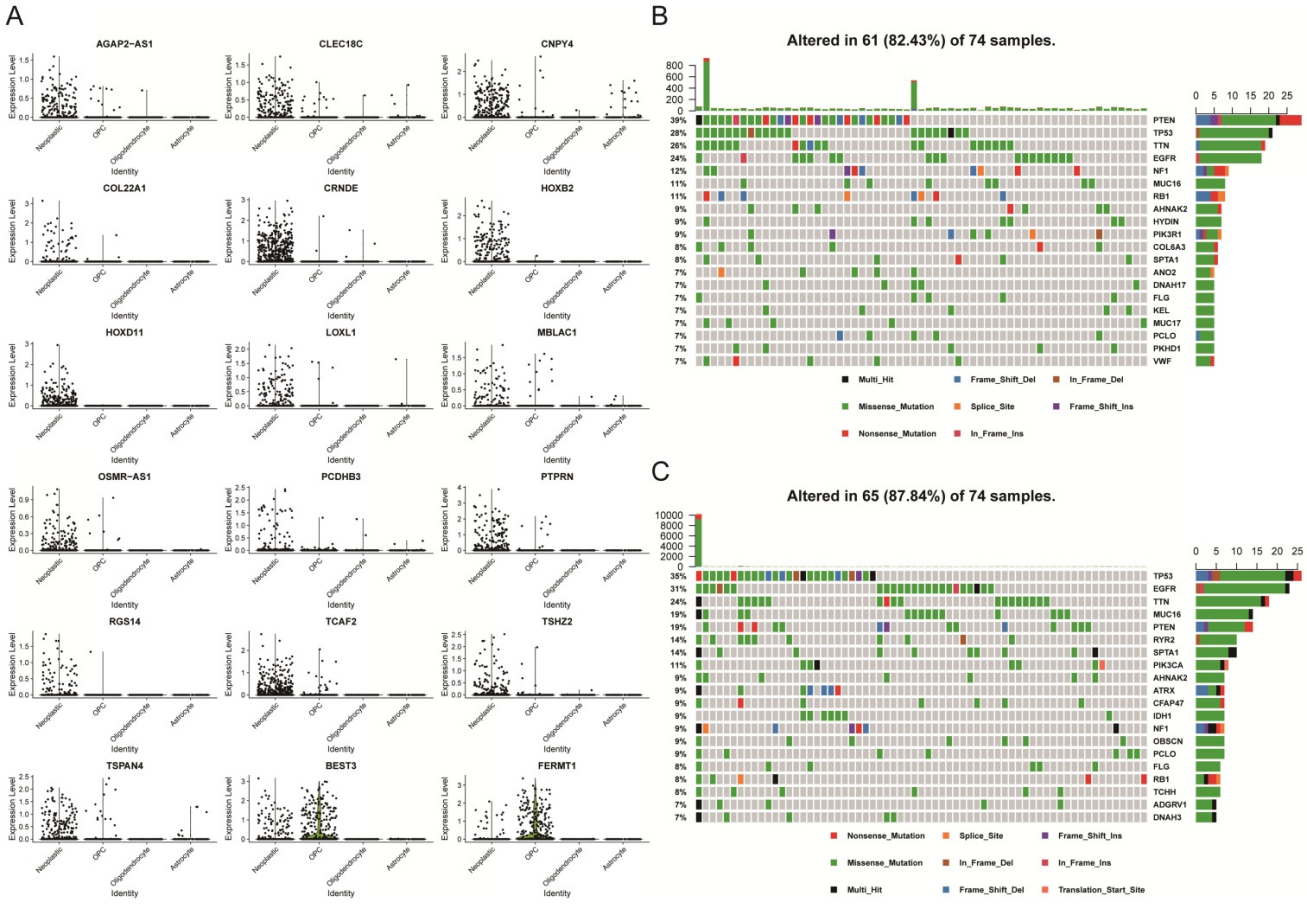
** Analysis of 18 prognostic genes in the single-cell and bulk analysis dataset. (A)** Differential expression levels of each gene in four clusters in the single-cell dataset. **(B)** The somatic mutation landscape of TCGA-GBM patients with a high PAS score. **(C)** The somatic mutational landscape of TCGA-GBM patients with a low PAS score.

After we identified 18 prognostic-associated signatures, we mapped the expression of the signatures to the UMAP-reduction plot, which was done in the single-cell dataset that was described above. It showed that two genes had lower expression levels in neoplastic cell clusters as compared to normal cells, while 16 genes displayed higher expression levels in the neoplastic cell cluster (Figure 5A and Figure S3). Specifically, five genes were involved in cell adhesion and migration, which included FERMT1, COL22A1, LOXL1, PCDHB3, and TCAF2. Four genes were transcription factor or involved in the regulation of transcription, like HOXB2, HOXD11, PTPRN, and TSHZ2. Moreover, we explored somatic mutation that was found when comparing the high with the low PAS score groups in the TCGA-GBM cohort.

The mutational landscape indicated that both groups had different mutation events, and the low PAS score group was found to have an increased frequency of mutation events (Figures 5B and 5C). In addition, patients with higher mutations might have an increased number of neoantigens that will increase the sensitivity of patients to chemotherapy, immunotherapy and targeted molecular therapy 25-27. Thus, these observations might provide a key reason to account for why low PAS score patients had improved prognostic outcomes.

### Development of a nomogram for predicting OS in GBM patients

**Figure 6:**
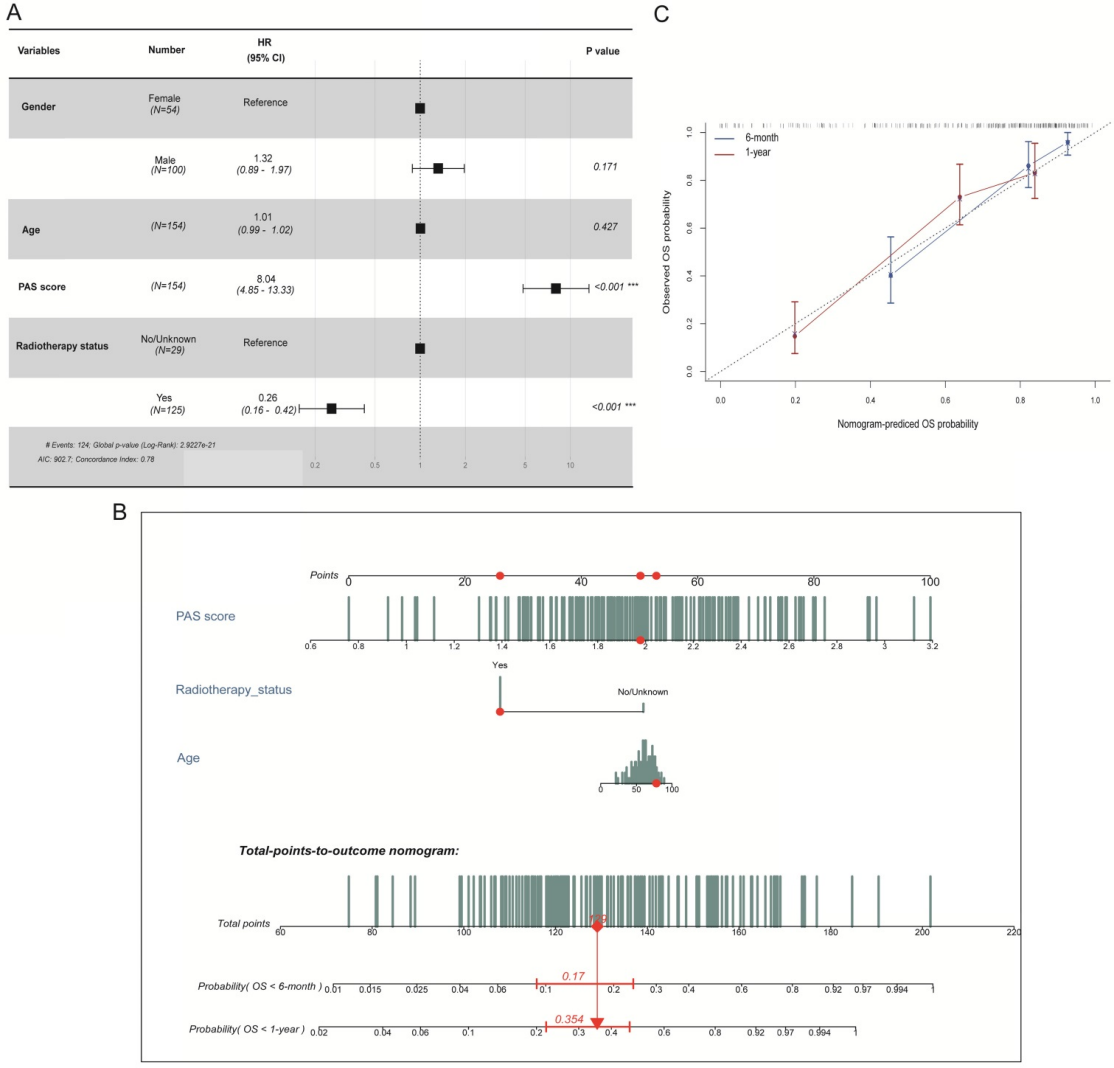
** Development of a nomogram in predicting the 6-month and 1-year survival probabilities in GBM patients. (A)** Multivariate Cox regression analysis of the PAS score with several clinical factors. **(B)** Integration of three factors to construct a nomogram for the prediction of 6-month and 1-year OS rates. **(C)** Calibration curve to validate the predictive efficacy of the model for 6 month and 1-year OS rates.

For the purpose of establishing a comprehensive model to predict the OS probability in GBM patients, we first analyzed an association between the PAS score and several clinical factors in the TCGA-GBM dataset. The hazard ratios for OS according to the PAS score, age, gender, and radiotherapy status were measured by multivariate Cox regression analysis (Figure 6A). It revealed that the PAS score and radiotherapy status were independent prognostic factors (p <0.001). Considering that the resistance of the human body to disease weakens with age, we also included age into the nomogram that was integrated with two independent factors.

The final three prognostic factors were utilized to develop a nomogram for predicting 6-month and 1-year OS probability (Figure 6B). A calibration curve was drawn to validate model efficacy (Figure 6C). Thus, the actual observed OS outcomes were approximately consistent with the predicted OS outcomes regardless of the 6-month or 1-year time frame, which showed an excellent working ability.

## Discussion

Bulk RNA-seq data from the TCGA-GBM dataset fails to accurately reflect the status of tumor and intra-tumorigenic heterogeneity 9, 10. This is in part due to the dominant cell cluster masking the transcriptomic characteristics of other cellular clusters. With the aid of single-cell RNA-seq datasets that were analyzed in this study, we compared and precisely discovered the DEGs between glioma cells and their original cells. These DEGs better describe the intra-tumorigenic heterogeneity of glioma, and provide us with a gene set for further analysis.

Furthermore, these DEGs indicated distinct molecular networks in glioma cells as compared to glia cells. GSEA analysis on DEGs revealed that up-regulated genes in glioma cells were enriched in the cell cycle, the cell division process and other tumor-associated pathways. By contrast, for normal cells, up-regulated genes were enriched in glial cell development-associated and synaptic function- associated pathways. KEGG enrichment analysis showed intriguing results as well, which were mostly enriched in pathways associated with lipid metabolism. Recent reports have shown that lipid metabolism is critical to glioma cells in terms of energy synthesis 28, 29, and fatty acids were shown to regulate the EGFR signaling pathway and to promote survival and proliferation of glioblastoma stem cells 24.

To connect the DEGs with prognosis, we first employed Cox regression and log-rank test analytics to preliminarily select genes with prognostic predicting potential, following which, we then employed variable selection analysis on the TCGA-GBM dataset. The final model included 18 genes that performed well on the dataset with a five-year OS AUC of 0.985.

Moreover, we used data from the GSE16011 cohort to test the generalization ability of the model, in which we found that accuracy was slightly decreased, and yet still achieved a five-year OS AUC of 0.842. In addition, patients that were divided by the PAS value displayed a distinct mutational landscape, which partially explained differences on OS. Demographic information that included age, and clinical information like chemotherapy were also good prognostic prediction factors, and thus we established a model using a nomogram to predict the overall survival of patients.

## Conclusion

We discovered a signature with only 18 genes by using a single cell RNA-seq dataset, from which we developed a PAS to evaluate patient prognoses. These genes characteristically represent the intra-tumor heterogenicity of glioblastoma. This signature has acceptable performance in terms of both the training and external datasets and showed acceptable generalization capabilities. This is a simple model with good performance characteristics that can be applied in the clinic. PAS is also associated with the somatic mutation profile of patients. These genes might act as potential prognostic biomarkers and provide useful clinical guidance to clinicians with the intent of developing personalized therapeutic schedules.

## Supplementary Material

Supplementary figures.

Supplementary table 1.

Supplementary table 2.

## Figures and Tables

**Table 1:** Statistical analysis of 18 genes associated with survival in the TCGA-GBM cohort.

Variables	Log-rank test	Univariate cox regression analysis		LASSO
	P value	HR (95% CI)	P value	Coefficient
AGAP2-AS1	0.018	1.24(1.10-1.40)	<0.001	0.057478
CLEC18C	0.012	3.73(1.58-8.80)	0.003	0.2067
CNPY4	0.046	1.87(1.32-2.65)	<0.001	0.054552
COL22A1	0.015	1.38(1.15-1.65)	<0.001	0.090813
CRNDE	0.010	1.31(1.04-1.66)	0.025	0.002454
HOXB2	0.001	1.19(1.05-1.34)	0.007	0.014237
HOXD11	0.049	1.32(1.06-1.64)	0.012	0.019821
LOXL1	0.011	1.43(1.21-1.69)	<0.001	0.034217
MBLAC1	0.006	1.79(1.18-2.73)	0.007	0.10079
OSMR-AS1	0.013	2.92(1.69-5.05)	<0.001	0.44677
PCDHB3	0.029	1.24(1.01-1.52)	0.039	0.01033
PTPRN	<0.001	1.42(1.21-1.67)	<0.001	0.19405
RGS14	0.006	1.57(1.18-2.07)	0.002	0.121256
TCAF2	<0.001	1.85(1.31-2.62)	<0.001	0.09673
TSHZ2	<0.001	1.93(1.34-2.78)	<0.001	0.023308
TSPAN4	0.015	2.11(1.40-3.18)	<0.001	0.0621
BEST3	0.037	0.73(0.59-0.91)	0.006	-0.0173
FERMT1	0.004	0.81(0.70-0.94)	0.006	-0.02211

## Abbreviations

GBM: glioblastoma
WHO: World Health Organization
OS: overall survival
RNA-seq: RNA sequencing
TCGA: The Cancer Genome Atlas
GEO: Gene Expression Omnibus
scRNA-seq: single-cell RNA sequencing
DEGs: differentially expressed genes
UCSC: University of California at Santa Cruz
FPKM: Fragments per kilobase of exon per million fragments mapped
UMAP: Uniform Manifold Approximation and Projection
OPCs: oligodendrocyte progenitor cells
GSEA: gene set enrichment analysis
GO: Gene Ontology
KEGG: Kyoto Encyclopedia of Genes and Genomes
PAS: prognostic associated signature
KM: Kaplan Meier
PAS score: prognostic associated signature score
LASSO: least absolute shrinkage and selection operator
ROC: receiver operating characteristic
AUC: areas under the curve
MAF: Mutation Annotation Format
PCA: principal component analysis
HR: hazard ratio
