# Supplementary material for: Improved Prognostic Prediction of Glioblastoma using a PAS Detected from Single-cell RNA-seq
Source: J Cancer. 2020 Apr 6;11(13):3751–61. doi: 10.7150/jca.44034 (PMC7171486; doi:10.7150/jca.44034)
Supplement: Supplementary file 1 — Supplementary figures. [file jcav11p3751s1.pdf]

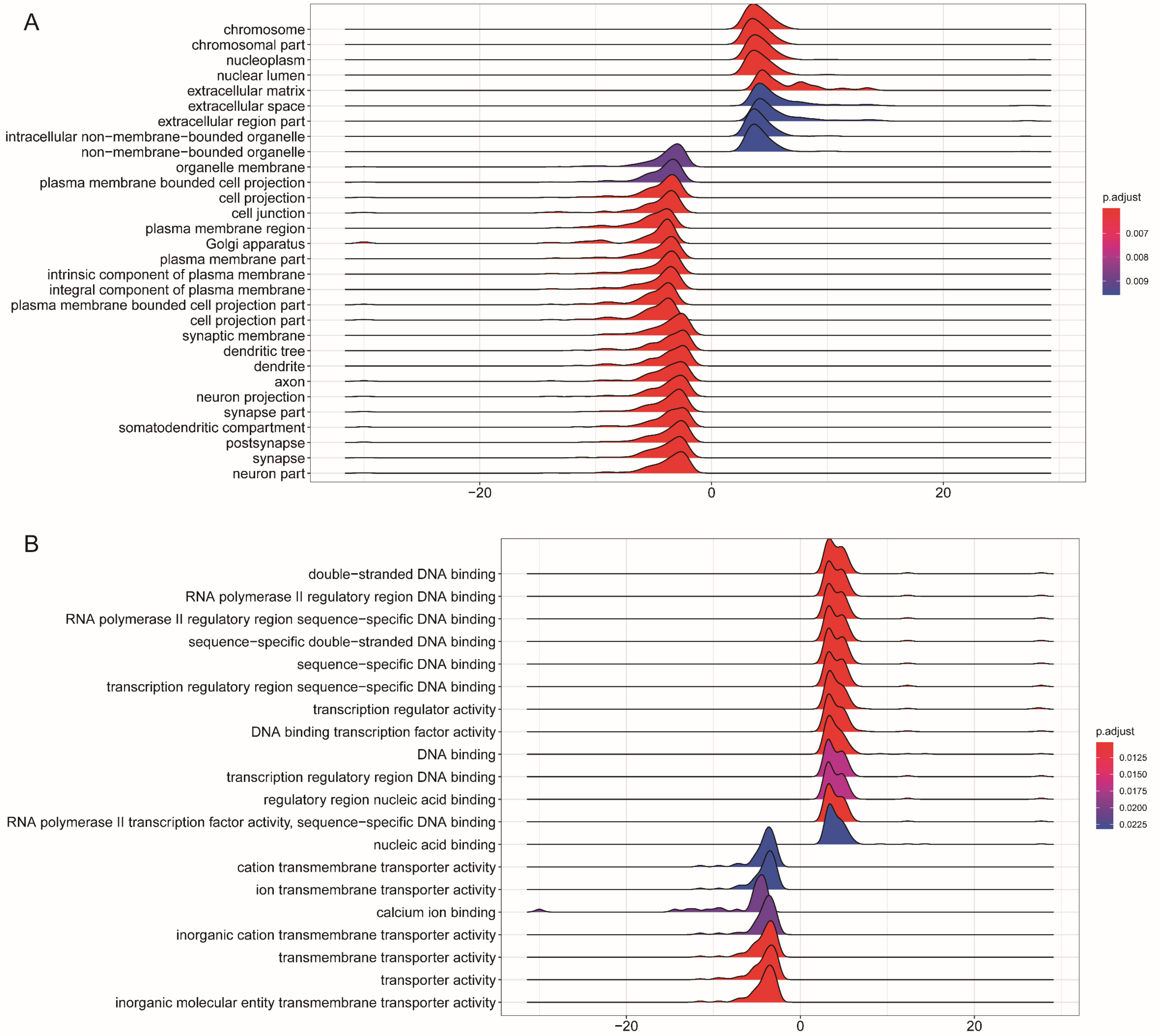

Figure S1. Additional GSEA results and KEGG analysis of differentially expressed genes. (A) The mostly significantly enriched cellular components of DEGs in the GSEA results, in which abscissa represents normalized enrichment score (NES). Rows with NES > 0 are results for tumor cells, otherwise for normal cells. (B) Same as in (A) but are enrichment analysis of molecular functions. (C) Diagram illustrating the top 10 pathways in the KEGG enrichment analysis of up-regulated genes. (D) Same as in (C) but for down-regulated genes.

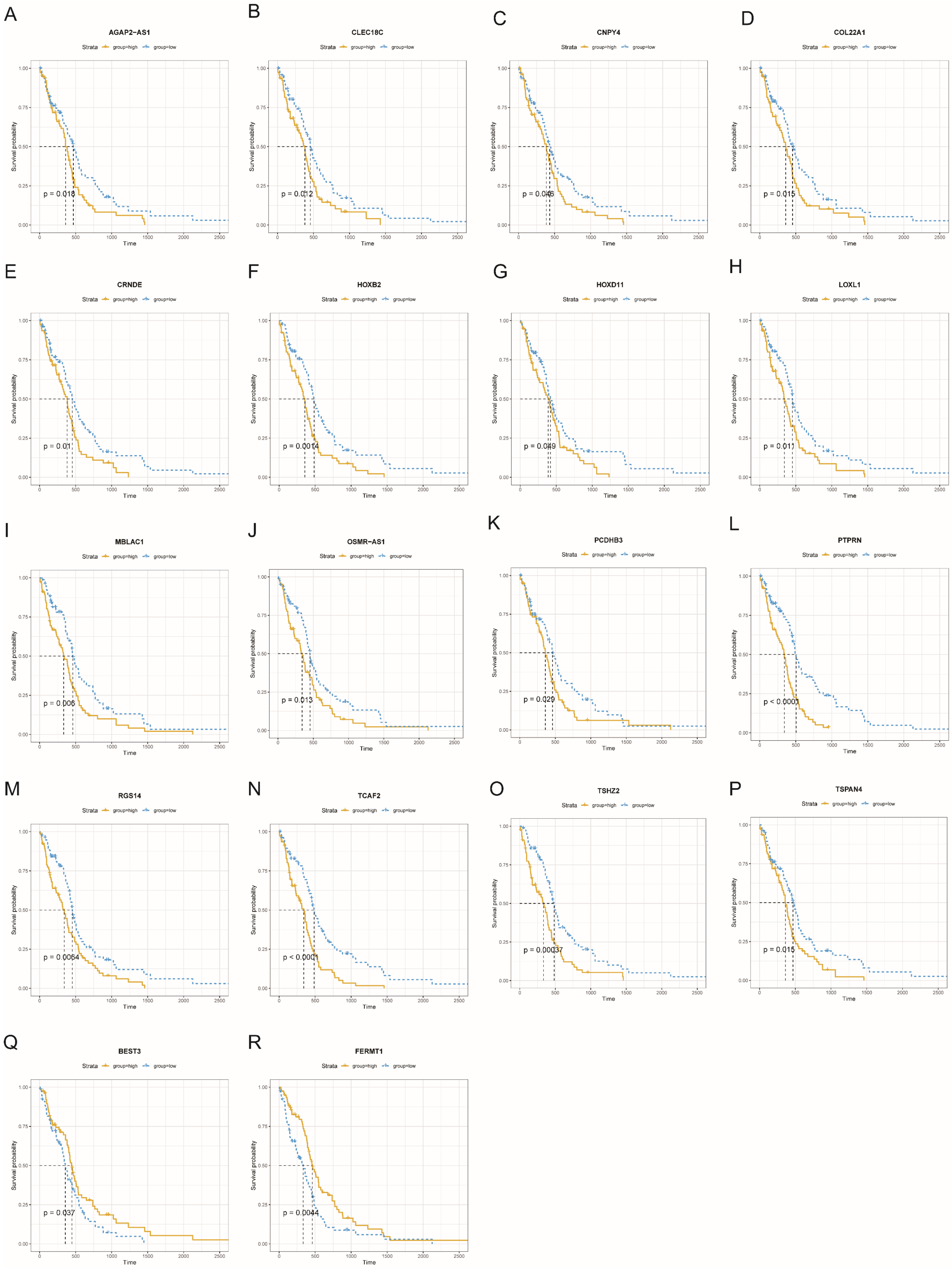

Figure S2. (A-R) KM curves of the 18 prognosis-associated genes in the TCGA-GBM cohort, where all genes correlated with the OS rate.

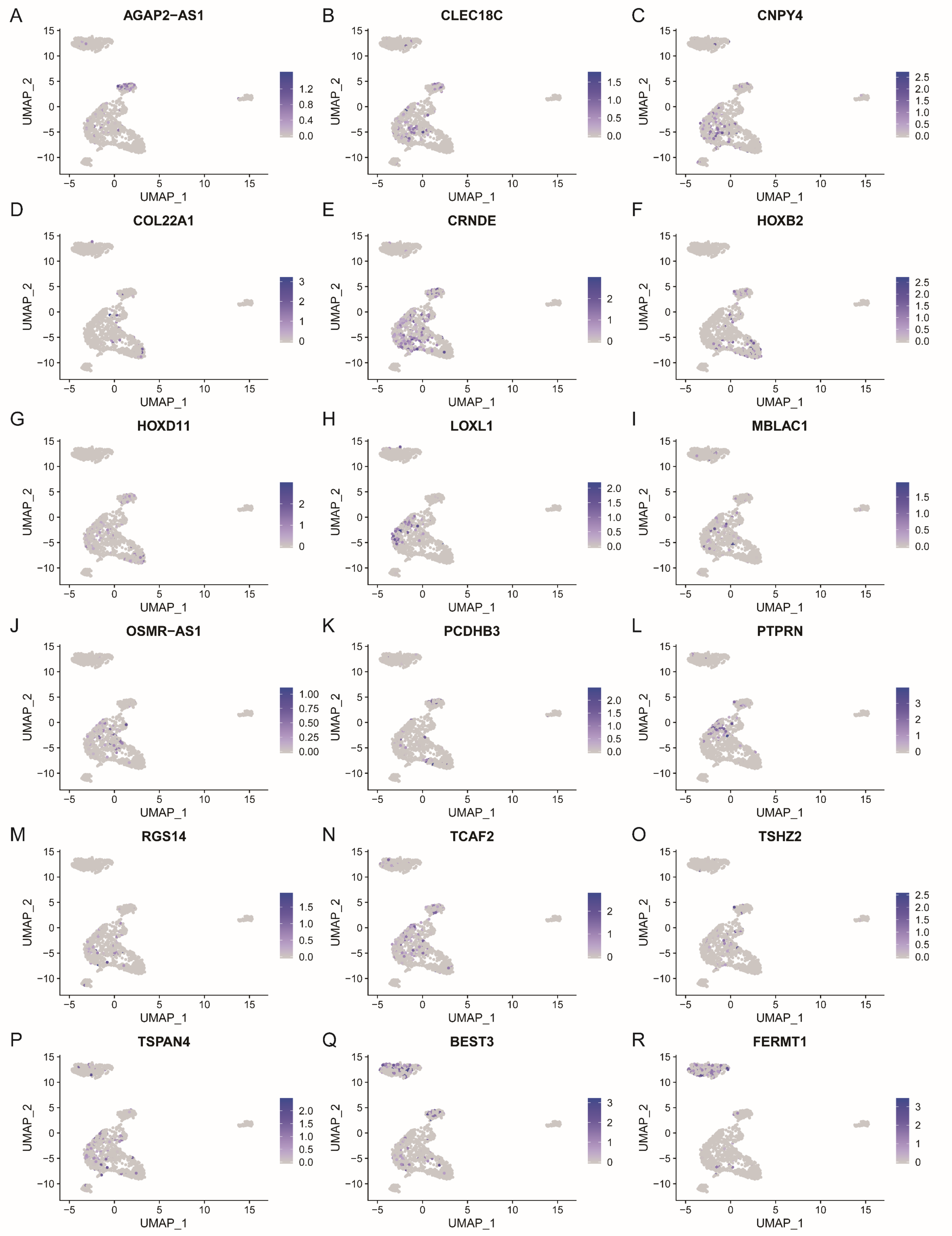

Figure S3. (A-R) Differential expression levels of 18 prognostic genes in four clusters in a single-cell RNA-seq via UMAP reduction.
